# Wolff-Parkinson-White syndrome type B

**DOI:** 10.11604/pamj.2022.43.177.32528

**Published:** 2022-12-05

**Authors:** Gwinyai Masukume, Mark Dixon

**Affiliations:** 1 Private Practice, Bulawayo, Zimbabwe; 2 National University of Science and Technology, Bulawayo, Zimbabwe

**Keywords:** Cardiology, electrocardiogram, Wolff-Parkinson-White syndrome

## Image in medicine

A 25-year-old female presented for a medical consultation complaining of sudden central chest pain, pleuritic in nature. She had never experienced such pain before. She denied experiencing coughing, shortness of breath or other respiratory or cardiac symptoms. Notably her grandparents, now in their 80s, were apparently being treated for ischemic heart disease attributed to their old age. She occasionally drank alcohol < 20 units per week. She was not using tobacco. On examination, her body mass index was 24.8 kg/m^2^, waist circumference 86 cm, pulse rate 59 beats per minute, blood pressure 103/68 mmHg, SpO_2_ 97% and temperature 37°C. Her general, cardiovascular and respiratory examinations were all normal. A resting electrocardiogram (ECG) showed a normal sinus rhythm, PR interval 126 ms, prolonged QRS duration 138 ms, and a delta wave (pictured - lead II). Leads I, II, and aVF had inverted T waves. Ventricular pre-excitation, Wolff-Parkinson-White syndrome type B was diagnosed. Echocardiography was unremarkable. The nature of her condition was explained to her. Advice to avoid medications like verapamil and digoxin was offered along with a recommendation to avoid over-strenuous activity. Screening ECGs were advised for her immediate family. As she had been asymptomatic up to this point, cardiology follow-up was recommended for consideration of a prophylactic ablation procedure. This case illustrates the benefit of a high index of suspicion and the utility of electrocardiography in diagnosing a rare condition.

**Figure 1:**
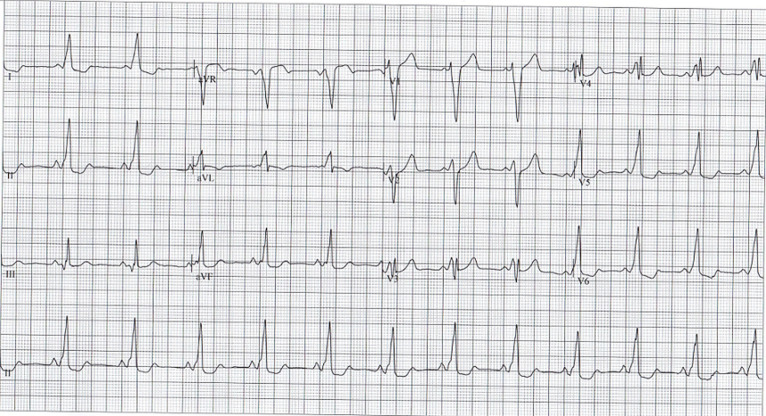
electrocardiogram showing a normal sinus rhythm, PR interval 126 ms, prolonged QRS duration 138 ms and a delta wave (pictured - lead II); leads I, II and aVF have inverted T waves

